# Dr. Charles A. Murray

**Published:** 1903-11

**Authors:** 


Dr. Charles A. Murray, one of the oldest and best-known physicians of Chemung county, died at his home in Van Etten, Thursday, September 3, 1903, aged 77 years. Dr. Murray graduated from Geneva Medical College in 1853, and practised his profession for fifty years, indeed, until within two weeks of the time of his death. He had been a member of Chemung County Medical Association, and was a prominent citizen in both business and politics.

